# Living Between Two Worlds: Reflections on Long‐Term Brain Tumour Survivorship From a Patient‐Researcher Perspective

**DOI:** 10.1111/hex.70957

**Published:** 2026-10-28

**Authors:** Julia Robertson

**Affiliations:** ^1^ Griffith University Nathan Queensland Australia

Cancer survivorship is often measured in years lived beyond diagnosis, treatment, or recurrence. Yet from a patient perspective, survivorship is not experienced as a number. Instead, it is lived through uncertainty, adaptation, relationships, loss, and the ongoing effort to construct a meaningful life alongside illness. Contemporary survivorship scholarship increasingly recognises that living with and beyond cancer extends beyond biomedical outcomes and involves psychological, social, and existential dimensions of experience. Cancer survivorship requires individuals to negotiate the realities of illness while continuing to participate in everyday life, integrating these experiences into a changing sense of self [1].

My experience of survivorship began with a brain tumour diagnosis in 2003 and was reshaped by a second diagnosis in 2023. Between those events, I completed higher education, developed a research career, became involved in advocacy and consumer engagement, and experienced multiple personal and family encounters with cancer. More than 20 years separated my diagnoses, but survivorship was not a period between two clinical events. It was an ongoing psychological process of adaptation.

One of the most enduring features of that process has been uncertainty. Illness uncertainty is recognised as a major psychosocial stressor in cancer care and may persist throughout the illness trajectory, including long after treatment has ended [2, 3]. Research demonstrates that uncertainty is associated with anxiety, depression, quality of life, and social support, and that interventions targeting uncertainty may improve psychosocial outcomes among survivors and family caregivers [2, 3].

For much of my life, uncertainty has existed in the background of major decisions. Career, relationships, and future planning have all been shaped by the reality that health cannot be taken for granted. The second brain tumour diagnosis in 2023 reinforced this experience. Familiarity with illness does not eliminate vulnerability. In my experience, long‐term survivorship did not make uncertainty disappear. Instead, it changed the way uncertainty was understood and negotiated.

From a psycho‐oncology perspective, this challenges assumptions that psychological adjustment follows a linear trajectory from diagnosis to recovery. My experience suggests that adaptation continues throughout survivorship and may be revisited as new health events emerge or life circumstances change. Taken together, these findings highlight the ongoing nature of psychological adjustment throughout survivorship [2, 3].

Alongside uncertainty, grief has been a recurring feature of survivorship. Within oncology, grief is often associated with bereavement. However, survivorship can also involve less visible losses, including certainty, independence, roles, future expectations, or aspects of identity. A qualitative systematic review of people affected by brain cancer identified experiences of distress, loneliness, identity disruption, role renegotiation, and unmet psychosocial support needs among both patients and family caregivers [4].

My own understanding of grief began before my first diagnosis. When I was 15 years old, my father was diagnosed with Hodgkin lymphoma, and my mother was diagnosed with malignant melanoma. During this period, I assumed caregiving responsibilities while supporting my two younger brothers. Becoming a caregiver before becoming a patient shaped how I later understood illness, vulnerability, and responsibility. It also informed my understanding of supportive care, adaptation, and family well‐being, experiences echoed in reports from patients and caregivers affected by brain cancer [4].

Over time, I have come to understand that grief and healing can coexist. Survivorship literature often emphasises resilience, adjustment, and post‐traumatic growth [5]. While these concepts are valuable, they can unintentionally suggest that distress should eventually resolve. My experience suggests otherwise. It has been possible to experience hope while also acknowledging loss, and to find meaning while continuing to carry sadness. Recognising this coexistence may provide a more nuanced understanding of survivorship than frameworks centred solely on recovery.

A second theme emerging from long‐term survivorship is identity. Cancer challenges assumptions about who we are, how we see ourselves, and how we relate to others [1]. Across more than two decades of survivorship, I have occupied multiple identities simultaneously: patient, survivor, former young carer, researcher, advocate, daughter, and family member. These identities have evolved across life stages and have not always aligned. Survivorship was not simply something I experienced. It became part of how I understood myself and my place in the world.

This identity work became particularly visible through my dual roles as survivor and researcher. As a psycho‐oncology researcher, I study questions relating to well‐being and survivorship. As a survivor, I have lived many of the experiences that form the focus of this work. This dual perspective has highlighted a disconnect between what is routinely measured and what survivors often perceive as most important.

Clinical outcomes remain essential. However, many experiences central to survivorship are less easily quantified. The cumulative burden of uncertainty, the psychological impact of caregiving, changes in identity and self‐understanding, and the search for meaning may be less visible within healthcare systems and research datasets than traditional clinical outcomes. Yet these experiences frequently shape how survivorship is lived.

Meaning‐making has become increasingly important within my own journey. Meaning‐making refers to the process through which individuals interpret challenging experiences and integrate them into broader understandings of themselves and their lives. A systematic review and meta‐analysis reported a significant association between meaning in life and post‐traumatic growth among people affected by cancer, highlighting the importance of meaning‐making processes in psychological adaptation [5].

For me, advocacy, consumer engagement, and research became ways of translating experiences of illness and loss into contribution and purpose. Importantly, meaning did not emerge because of illness. Rather, it emerged through ongoing efforts to respond to illness.

More than 20 years separated my first and second brain tumour diagnoses. From a psycho‐oncology perspective, survivorship may be best understood not as a destination reached after treatment but as a continuing process of negotiating uncertainty, integrating loss, adapting to changing identities, and creating meaning across the lifespan [1, 2, 3, 5]. Expanding our understanding of survivorship in these terms may help move the field beyond measuring survival and towards understanding how people adapt, create meaning, and live with and beyond cancer.

## Author Contributions

**Julia Robertson:** conceptualisation, writing – original draft, writing – review and editing.

## Funding

The author has nothing to report.

## Conflicts of Interest

The author declares no conflicts of interest.

## Data Availability

Data sharing not applicable to this article as no datasets were generated or analysed during the current study.

## References

[1] B. Zebrack , “Cancer Survivorship: A Framework for Quality Cancer Care,” JNCI: Journal of the National Cancer Institute 116, no. 3 (2024): 352–355, 10.1093/jnci/djad266. 38113418

[2] T. Guan , Y. Qan'ir , and L. Song , “Systematic Review of Illness Uncertainty Management Interventions for Cancer Patients and Their Family Caregivers,” Supportive Care in Cancer: Official Journal of the Multinational Association of Supportive Care in Cancer 29, no. 8 (2021): 4623–4640, 10.1007/s00520-020-05931-x. PMC8236440 33495851

[3] T. Guan , M. V. Chapman , L. de Saxe Zerden , A. Sharma , D. G. Chen , and L. Song , “Correlates of Illness Uncertainty in Cancer Survivors and Family Caregivers: A Systematic Review and Meta‐Analysis,” Supportive Care in Cancer: Official Journal of the Multinational Association of Supportive Care in Cancer 31 (2023): 242, 10.1007/s00520-023-07705-7. 36977804

[4] C. Paterson , C. Roberts , J. Li , et al., “What Are the Experiences of Supportive Care in People Affected by Brain Cancer and Their Informal Caregivers: A Qualitative Systematic Review,” Journal of Cancer Survivorship 18, no. 5 (2024): 1608–1629, 10.1007/s11764-023-01401-5. PMC10229398 37256499

[5] M. Almeida , C. Ramos , L. Maciel , M. Basto‐Pereira , and I. Leal , “Meaning in Life, Meaning‐Making and Posttraumatic Growth in Cancer Patients: Systematic Review and Meta‐Analysis,” Frontiers in Psychology 13 (2022): 995981, 10.3389/fpsyg.2022.995981. PMC9784472 36570997

